# Bridging the gap: how patient-derived lung cancer organoids are transforming personalized medicine

**DOI:** 10.3389/fcell.2025.1554268

**Published:** 2025-04-15

**Authors:** Chaoxing Liu, Chao Shi, Siya Wang, Rong Qi, Weiguo Gu, Feng Yu, Guohua Zhang, Feng Qiu

**Affiliations:** ^1^ Department of Oncology, Gaoxin Branch of the First Affiliated Hospital of Nanchang University, Nanchang, Jiangxi, China; ^2^ Nanchang Key Laboratory of Tumor Gene Diagnosis and Innovative Treatment Research, Gaoxin Branch of the First Affiliated Hospital of Nanchang University, Nanchang, Jiangxi, China; ^3^ Department of Oncology, The First Affiliated Hospital of Nanchang University, Nanchang, Jiangxi, China

**Keywords:** lung cancer, organoids, cultivation, clinical applications, transforming

## Abstract

Lung cancer is a major malignancy that poses a significant threat to human health, with its complex pathogenesis and molecular characteristics presenting substantial challenges for treatment. Traditional two-dimensional cell cultures and animal models are limited in their ability to accurately replicate the characteristics of different lung cancer patients, thereby hindering research on disease mechanisms and treatment strategies. The development of organoid technology has enabled the growth of patient-derived tumor cells in three-dimensional cultures, which can stably preserve the tumor’s tissue morphology, genomic features, and drug response. There have been significant advancements in the field of patient-derived lung cancer organoids (PDLCOs), challenges remain in the reproducibility and standardization of PDLCOs models due to variations in specimen sources, subsequent processing techniques, culture medium formulations, and Matrigel batches. This review summarizes the cultivation and validation processes of PDLCOs and explores their clinical applications in personalized treatment, drug screening after resistance, PDLCOs biobanks construction, and drug development. Additionally, the integration of PDLCOs with cutting-edge technologies in various fields, such as tumor assembloid techniques, artificial intelligence, organoid-on-a-chip, 3D bioprinting, gene editing, and single-cell RNA sequencing, has greatly expanded their clinical potential. This review, incorporating the latest research developments in PDLCOs, provides an overview of their cultivation, clinical applications, and interdisciplinary integration, while also addressing the prospects and challenges of PDLCOs in precision medicine for lung cancer.

## 1 Introduction

In recent years, although clinical treatments for lung cancer have advanced due to improved surgical techniques, radiation therapy, refined tumor staging, and continuously updated molecular and genetic classifications, it remains a leading global health threat (Sung et al., 2021) (Zheng et al., 2023). Lung cancer is categorized into non-small cell lung cancer (NSCLC) and small cell lung cancer (SCLC), with NSCLC accounting for approximately 85% of all lung cancer cases. Lung adenocarcinoma (LUAD) and squamous cell carcinoma (LUSC) each representing around 50% of NSCLC cases (Herbst et al., 2008). The treatment of advanced LUAD has evolved from initial platinum-based chemotherapy to first-line targeted therapies targeting driver gene mutations (EGFR, ALK, ROS1, NTRK1, BRAFV600E, KRAS, and RET), which have become the standard treatment for driver gene mutation-positive LUAD patients. For patients with wild-type driver genes, immune checkpoint inhibitors combined with anti-angiogenesis therapies and/or platinum-based chemotherapy are commonly used (Ettinger et al., 2022). However, the tumor phenotype in patients are likely to change as the disease progresses and during treatment. This is primarily due to the tumor microenvironment (TME), which influences tumor cell phenotypes through the secretion of soluble signals and complex interactions between cells and the extracellular matrix (ECM) (Qian et al., 2020). Additionally, numerous studies have shown that genomic and transcriptomic instability leads to alterations in epigenetic, metabolic, and morphological characteristics, which in turn result in distinct cellular subpopulations within tumors and among individuals (Turajlic et al., 2019; Marusyk et al., 2020). The heterogeneity represented by these subpopulations is one of the major reasons for the failure of current precision therapies in lung cancer patients (Dagogo-Jack and Shaw, 2018). Therefore, developing models that can accurately replicate the biological and genetic heterogeneity between and within tumors in lung cancer patients is crucial for exploring disease and resistance mechanisms. Ultimately, this will facilitate the design of personalized precision treatment strategies, which undoubtedly holds revolutionary transformative potential.

The advent of organoid technology has made it possible to culture patient-derived primary lung cancer cells in a three-dimensional (3D) *in vitro* system. Organoids were first reported in 1946 as a synonym for teratoma in the field of oncology (Smith and Cochrane, 1946). In 1993, Benali et al. (1993) described the 3D self-organizing structures of adult airway epithelium for the first time. With continuous development, in 2009, Professor Hans Clevers reported a novel intestinal organoid culture system, where the cultured organoids largely replicated the *in vivo* intestinal tissue structure and contained relatively intact stem cells (Sato et al., 2009). Since then, organoid technology has rapidly advanced in the biomedical field, with organoid models derived from various human tissues emerging, including colon cancer (Engel et al., 2020; Zhao et al., 2020), pancreatic cancer (Driehuis et al., 2019), breast cancer (Mazzucchelli et al., 2019), liver cancer (Qin et al., 2020; Cao et al., 2019), ovarian cancer (Maru et al., 2019), bladder cancer (Mullenders et al., 2019; Lee et al., 2018), prostate cancer (Gao et al., 2014), head and neck squamous cell carcinoma (Driehuis et al., 2020), normal airway epithelium organoids (AOs) (Lu et al., 2021), and lung cancer organoids [LUAD (Li et al., 2020a), LUSC(Kim et al., 2019), SCLC (Choi et al., 2021), large cell carcinoma (Shi et al., 2020), and adeno-squamous carcinoma (Kim et al., 2019)].

**FIGURE 1:**
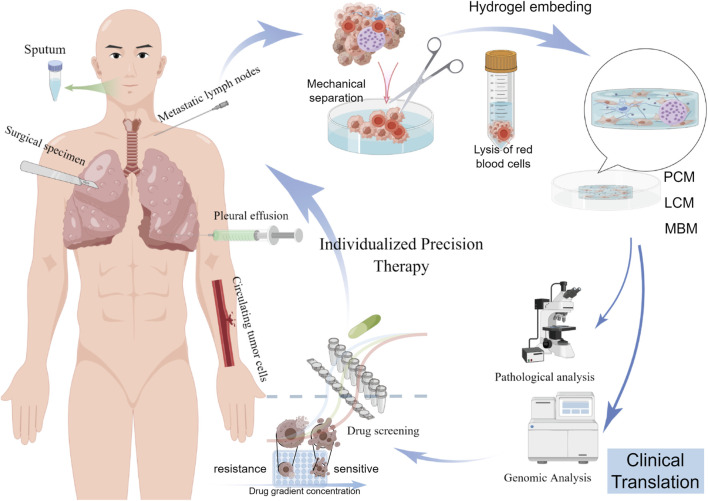
Flowchart illustrating the establishment and application of PDLCOs in individualized precision therapy. The process involves isolating cancer cells from various tissue (sputum, surgical specimens, metastatic lymph nodes, pleural effusion, and circulating tumor cells), embedding them in hydrogel for culture, forming PDLCO models, followed by pathological analysis, genomic analysis, and drug screening to develop personalized treatment strategies. PCM: permissive culture medium; LCM: limited culture medium; MBM: minimum basal medium (Supplementary Table S1).

Compared with traditional two-dimensional (2D) cell lines and animal models, organoids offer unique advantages. The cultivation of 2D cell lines is the most widely used method for cancer modeling, with a large number of lung cancer cell lines and related genomic information cataloged in the Cancer Cell Line Encyclopedia (Gillet et al., 2013) (Barretina et al., 2012). However, although these immortalized 2D cell lines grow rapidly and are relatively easy to culture, they fail to replicate the complex interactions between tumor cells, the surrounding ECM, and the components of the tumor immune microenvironment (LeSavage et al., 2022). While patient-derived xenografts (PDX) and genetically engineered models partially reflect the 3D nature of tumor tissues (Lai et al., 2017), they require large amounts of primary tumor tissue to be transplanted into immunodeficient mice and suffer from low transplantation success rates, technical challenges, long experimental periods, high costs, and limited suitability for large-scale drug screening (Day et al., 2015; Ben-David et al., 2017; Morgan et al., 2017; Moro et al., 2012) (Table1). Since the first report of a personalized patient-derived lung cancer organoids (PDLCOs) model in 2017 (Pauli et al., 2017), PDLCOs have now been successfully cultured from a variety of patient-derived specimens, including surgical samples, biopsies, pleural effusions, sputum, and circulating tumor cells (Figure 1).

**TABLE 1:** Comparison of PDLCOs, cell lines, and PDX models.

Characteristic	PDLCO	Cell line	PDX
Difficulty of operation	**	*	***
Cost	**	*	***
Passaging	**	*	***
Cycle	**	*	***
Success rate	**	***	*
Reproduction tumor heterogeneity	***	*	***
Reproduction of genetic characteristics	***	*	***
Clinical prediction	***	*	**
High-throughput drug screening	***	***	*
Tumor Microenvironment	***	-	**
Cross-disciplinary application	***	*	*

*represents the degree of implementation,*Low, **Medium, ***High.

This review focuses on the methods, processes, and conditions for culturing PDLCOs, exploring their rapid development and potential applications in personalized lung cancer efficacy prediction, drug screening after resistance, organoid biobank construction, and drug development. Moreover, PDLCOs serve not only as a bridge between basic research and clinical translation but also integrate with cutting-edge technologies such as tumor assembloid, artificial intelligence (AI), 3D bioprinting, organoid-on-a-chip, gene editing, and single-cell sequencing (scRNA-seq), thereby advancing our understanding of lung cancer initiation, progression, and treatment. Although organoid technology has provided new perspectives for lung cancer research, certain limitations remain. This review discussed these challenges and propose strategies to improve the translation of PDLCOs technology, aiming to maximize its potential from basic research to clinical applications in lung cancer.

## 2 Construction and validation of PDLCOs

### 2.1 Source and culturing process of PDLCOs samples

For early-stage operable patients, PDLCOs are typically cultured using post-surgical cancer and adjacent normal tissue samples. It is important to remove excess fat and necrotic tissue from the post-surgical tumor samples to enhance the purity of the PDLCOs (Guan and Huang, 2022). For patients with advanced, inoperable cancer, tissue samples are obtained through image-guided biopsy, which serves both for diagnosis and PDLCOs culture (Choi et al., 2021). Additionally, advanced lung cancer patients often present with pleural or pericardial effusion (Wang et al., 2023). After performing a thoracentesis to relieve symptoms, some of the pleural effusion can be sent for pathological examination, while the remaining samples can be used for clinical research (Wahidi et al., 2017). Numerous studies have demonstrated that tumor cells from pleural effusion can be successfully cultured into PDLCOs (Wang et al., 2023; Mazzocchi et al., 2019; Yokota et al., 2021; Kim et al., 2021; Ma X. et al., 2021; Surina et al., 2023). Furthermore, PDLCOs have been successfully cultured from circulating tumor cells (CTCs) in the blood, sputum, and metastatic tumor samples from subcutaneous tissue and bone (Ebisudani et al., 2023) (Lee et al., 2020). Overall, most pathological diagnostic samples are suitable for culturing PDLCOs, and even human-induced pluripotent stem cells (hiPSCs) can be used to obtain the required samples (Wong et al., 2012). Compared with organoids derived from other types of tumors, PDLCOs present unique characteristics and challenges during their establishment and culture. For instance, gastrointestinal cancer organoids often require heightened awareness of microbial contamination due to the presence of commensal microorganisms (Marinucci et al., 2021; Nam et al., 2021), while liver cancer organoids must address the risk of hepatitis virus invasion (Song et al., 2023). In contrast, PDLCOs have more diverse sample sources and lower risks of pathogenic microbial infections. However, special attention should be given to external contamination, such as ash or dust particles, during the culture process. Notably, for patients with malignant pleural effusion due to advanced lung cancer, the routine administration of chemotherapeutic drugs via intrapleural perfusion can significantly reduce the viability of primary tumor cells and decrease the success rate of organoid cultivation (Yi et al., 2016). Therefore, it is essential to be familiar with the patient’s medical history and treatment regimen prior to initiating organoids culture. The key to successful PDLCOs culture lies in the viability of the cancer cells, particularly the number and quality of the “seed” cells, which is the critical first step for the successful growth of PDLCOs.

**FIGURE 2:**
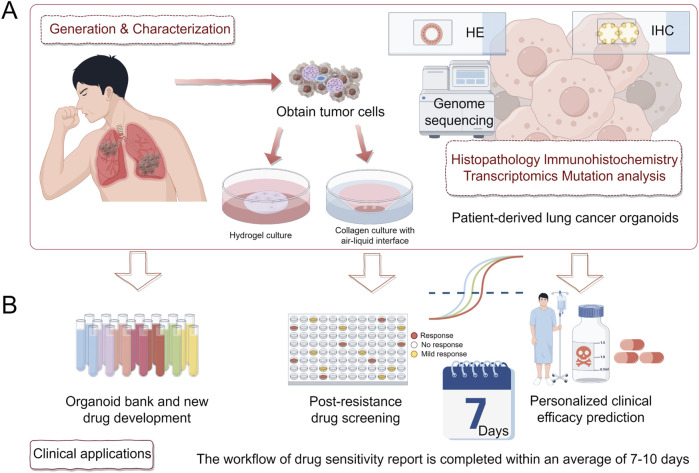
Generation, validation, and clinical applications of PDLCOs. **(A)**. Tumor cells are isolated from patient samples and cultured using hydrogel or collagen with an air-liquid interface. PDLCOs are characterized through histopathology, immunohistochemistry, transcriptomics, and mutation analysis. **(B)**. Clinical applications include the establishment of an organoid biobank and new drug development, post-resistance drug screening, and personalized clinical efficacy prediction.

After obtaining tumor tissue or primary culture samples, they should be immediately stored at a low temperature (4°C) for subsequent processing, such as digestion and red blood cell lysis (Li Z. et al., 2020). The resulting primary cells are resuspended in Matrigel, and after allowing the Matrigel to solidify (approximately 30 min), the cells are submerged in PDLCOs culture medium for 3D cultivation (Sato et al., 2009). This method is widely used in most laboratories (Acosta-Plasencia et al., 2024; Kang et al., 2023; Lee et al., 2024). An alternative method involves culturing PDLCOs using the air-liquid interface (ALI) technique (Ekanger et al., 2025). Specifically, minced tumor tissue (less than 1 mm^3^) is resuspended in collagen matrix and placed on pre-solidified collagen in a small chamber to form a bilayer air-liquid culture system. After the gel has formed, it is transferred to an external culture dish containing the medium and incubated at 37°C (Neal et al., 2018). Neal et al. (2018) successfully cultured organoids from NSCLC patients using this method and reported that these organoids retained certain functional features and aspects of the original TME. However, the ALI method is technically challenging and has a relatively low success rate (Yaqub et al., 2022). Currently, both of the aforementioned organoid derivation strategies have been successful in establishing PDLCOs (Figure 2A). However, due to slight methodological differences across laboratories, achieving standardized culture remains difficult, which limits the broader application of PDLCOs research (Vella et al., 2024). The following sections of this review will primarily focus on the use of the first immersion embedding method for PDLCOs cultivation.

After seeding primary lung cancer cells for organoid culture, passage can be performed when the actual growth rate of organoids reaches approximately 7–10 days or when more than 30% of lung cancer organoids exceed a diameter of 200 μm (Li Z. et al., 2020; Kim et al., 2019). The specific procedure involves incubating the organoids in pre-warmed TrypLE Express at 37°C for 5–10 min to fully dissociate them into a single-cell suspension, followed by re-embedding in fresh Matrigel for continued culture. Notably, to maintain genomic stability during long-term passaging, it is recommended to perform regular whole-exome sequencing (WES) to systematically screen and select high-quality organoid clones with stable genetic traits, ensuring experimental reliability (Dijkstra et al., 2020) (Dijkstra et al., 2018).

### 2.2 Matrigel and culture medium for PDLCOs cultivation

Organoids are typically supported by ECM scaffolds derived from animal tissues, with the most commonly used being Matrigel extracted from mouse sarcoma (Orkin et al., 1977). The main ECM proteins in Matrigel include laminin (approximately 60%) and type IV collagen (approximately 30%), which provide essential structural and nutritional support to the cells (LeSavage et al., 2022). Hydrogel has characteristics such as tunable mechanical stiffness, uniform microstructure, and pore permeability (Liu et al., 2018). Changes in the stiffness of the hydrogel can influence its microstructure and 3D permeability, thereby affecting the vitality of the organoids (Cha et al., 2010). Currently, commonly used hydrogels for organoid culture can be categorized into naturally derived hydrogels and engineered synthetic hydrogels (see Supplementary Table S2). Given the animal-derived nature of Matrigel, this study classifies it as a naturally derived hydrogel. While commercialized Matrigel supports PDLCOs culture, its animal-derived nature introduces batch-to-batch variability and potential xenogeneic impurities, which may impact the phenotype and growth of PDLCOs (Aisenbrey and Murphy, 2020). Therefore, improving the source of the matrix gel is an urgent issue. Engineered synthetic matrices, in contrast to those derived from animals, provide greater batch-to-batch consistency, which enhances the reproducibility and standardization of PDLCOs generation and culture (LeSavage et al., 2022). Additionally, polymorphic hydrogel with well-defined compositions (Zhou et al., 2009) and plant-derived matrix gels (Li T. et al., 2024) are promising alternatives for future PDLCOs cultivation, as they may better simulate the physical forces acting on lung cancer cells during respiratory processes. Standardizing the establishment of matrices that closely mimic the ECM components and structures of lung cancer patients is essential for better understanding cancer development.

The initial culture medium for PDLCOs was based on a colon cancer organoid system established in 2009. This system incorporated key growth factors, such as FGF7 and FGF10, which are typically expressed in normal lung tissue, into the medium. This formulation, known as the AOs culture medium, achieved a success rate of 28% (Sachs et al., 2019). Currently, PDLCOs are cultured in an advanced Dulbecco’s Modified Eagle Medium (adDMEM/F12) as the base medium (Orkin et al., 1977). Their growth is regulated by four fundamental signaling pathways: (1) activation of the Wnt/β-catenin pathway, which maintains stem cell pluripotency; (2) activation of the TGF-β/Smad pathway, which facilitates stem cell differentiation; (3) the p38 MAPK pathway, which induces cell death, with SB202190 protecting cells from apoptosis by inhibiting this pathway; (4) the ROCK pathway, which mediates anoikis in cells (Ma et al., 2022). The growth factors added to the PDLCOs culture medium play either a promoting or inhibitory role in these signaling pathways:1) Supplements related to cell survival (such as B27, N-acetylcysteine (NAC), Nicotinamide, Y-27632, and SB202190); 2) Supplements related to stemness/differentiation balance (such as WNT/R-spondins, Noggin, and the FGF family); 3) Supplements related to cell proliferation (such as EGF, A38-01); 4) Other supplements (such as N2, CHIR99021, Hepatocyte Growth Factor (HGF),Prostaglandin E2 (PGE2), Nutlin-3a, Gastrin 1, forskolin, and heregulinβ-1) (Ma et al., 2022). However, different laboratories have made varying degrees of improvements to the PDLCOs culture system, resulting in significant differences in success rates (Supplementary Table S1). This may be due to the complex genetic backgrounds of lung cancer patients, and after prolonged disease progression and multi-line treatments, the TME and epigenetic characteristics change. Therefore, a single culture medium formula may not meet the growth and differentiation needs of lung cancer cells from various heterogeneous backgrounds. Thus, the standardization of the culture process, along with grouping patients based on their genetic and treatment backgrounds to provide targeted culture medium formulas, is particularly crucial.

### 2.3 Validation of PDLCOs

The purity of PDLCOs cultures and their high fidelity to parental tumor samples are essential for establishing organoid models and conducting subsequent translational. Therefore, after establishing *in vitro* PDLCO models, it is necessary to perform multi-level validation, including histological examination, immunohistochemistry (IHC), Immunofluorescence (IF), genomic analysis, and even validation at the patient-derived xenograft organoids level (Kim et al., 2019; Shi et al., 2020; Dijkstra et al., 2020), in order to prevent contamination by AOs and avoid genetic alterations. Initially, hematoxylin and eosin (H&E) staining can be used to compare the cellular morphology and tissue characteristics of PDLCOs with those of parental tumor tissues. Subsequently, the fidelity of the PDLCOs can be further validated through the expression of characteristic pathological markers and even genomic sequencing. Common IHC markers for LUAD (whether solid or cystic) include NapsinA, Cytokeratin 7(CK7), and Thyroid Transcription Factor-1 (TTF-1); for adeno-squamous carcinoma, markers include p63, CK7, and CK5/6; for LUSC, markers include p63, CK5/6, and p40; and for SCLC, markers include CD56 and Synaptophysin (Kim et al., 2019; Dijkstra et al., 2020). Additionally, samples from PDLCOs or lung cancer tissues can be analyzed using WES, whole genome sequencing (WGS), sanger sequencing, high-throughput genomic sequencing, droplet digital PCR, or PCR-based methods to detect mutational hotspots. According to genomic sequencing data from PDLCOs, Kim et al. reported that short-term cultured PDLCOs retained 92.7% of the gene mutations present in the original tumor tissue (Kim et al., 2019; Kim et al., 2021). However, some PDLCOs exhibit additional gene mutations not detected in the original tissue, potentially due to cell cross-contamination, limitations in genetic testing methods, or the presence of low-frequency mutations in the primary tumor. In contrast, long-term passaged (over 10 passages) PDLCOs maintain the overall mutation profile and copy number variations of the parental tumor, although approximately 80% of long-term cultured PDOs show an increased frequency of sub-clonal mutations (Li Z. et al., 2020; Kim et al., 2019; Choi et al., 2021; Shi et al., 2020; Kim et al., 2021). The clinical and research value of PDLCOs, including their use in drug screening, can only be realized when it is confirmed that these organoids consistently reflect the genomic and histological characteristics of the original tumor tissue.

## 3 Clinical applications of PDLCOs

The advent of molecular targeted therapies and immunotherapies has significantly transformed the treatment landscape of lung cancer over the past decade. In clinical practice, treatment decisions for advanced lung cancer patients are typically based on histological subtypes and/or genetic mutation profiles. However, even within the same pathological subtype, there remains considerable heterogeneity among individual lung cancer patients. Subgroups of patients with the same driver mutations also exhibit a wide range of molecular diversity. This heterogeneity may lead to varying clinical presentations and differential responses to anticancer treatments (Skoulidis and Heymach, 2019). As a result, achieving fully personalized precision medicine for lung cancer patients remains a challenge. PDLCOs are increasingly being used in various areas such as personalized clinical efficacy prediction, drug screening for post-resistance, organoid biobank construction, and drug development (Figure 2B), contributing to propel the advancement of precision diagnostics and treatment for lung cancer patients (Table 2).

**TABLE 2:** Comprehensive overview of oncology therapeutic interventions and biomarker-driven responses.

Study category	Biological specimen	Therapeutic approach	Mechanistic insight/Clinical outcome	Ref. PMID
Targeted Therapy Resistance	Post-resistance re-biopsy samples	Tivantinib (MET D1228N mutation)	Overcame 3rd-gen EGFR-TKI resistance	36806896
Drug Sensitivity Prediction	Malignant pleural effusion	Cancer organoid-based diagnosis reactivity prediction (CODRP) platform predicts EGFR-TKI efficacy	Complete drug sensitivity classification within 10 days, which is highly consistent with clinical efficacy	38773241
Pathway-targeted Therapy	Multi-source samples	Wnt-targeted therapy	NKX2-1 status stratifies sensitivity to Wnt-targeting therapy. EGFR wild-type PDLCOs are more sensitive to Wnt ligand inhibitors	36870059
Dual-target Treatment	Malignant effusion	EGFR+/METamp + combination	Dual-targeting showed potent anticancer activity	35437920
Platinum Resistance Reversal	Platinum-resistant biopsy	Arginine combined with cisplatin	Inhibition of Hedgehog pathway enhances platinum sensitivity	36034794
Targeted Therapy Resistance	Surgical resection samples	DCLK1 inhibitor combined with osimertinib	Inhibition of the Wnt/β-catenin pathway can reverse EGFR-TKI resistance	35157971
Platinum Resistance Reversal in LUSC	Malignant effusion	Platinum chemotherapy combined with Halofuginone	PI3K/AKT and MAPK pathway inhibition enhances platinum sensitivity	34901018
Natural Compound Screening	Surgical resection samples	Berberine (95% CI, 0.092–1.55 μM)	In the sensitivity test of natural compounds, PDO is more sensitive than cell lines	31973535
Immuno-combination Therapy	Biopsy	PD-L1 inhibitor combined with MEK inhibitor	Dual blockade significantly enhances anti-tumor activity	31196138
Targeted Therapy Resistance	Biopsy	Long-term exposure to erlotinib	Inducing BRAF V600E/KRAS G12D and other co-mutations	32898185
Rare target mutations	Surgical resection specimen (HER20 exon insertion mutations)	Verify the efficacy of pyrotinib against HER2 exon 20 insertion mutations	The drug sensitivity results of PDLCOs were completely consistent with clinical observations (ORR 100%)	30596880

### 3.1 Personalized clinical efficacy prediction in lung cancer

Personalized treatment for lung cancer patients relies on a clear genetic profile, enabling clinicians to make targeted therapeutic decisions. PDLCOs provide a wealth of personalized features that reflect the genomic and proteomic characteristics of primary tumor cells, thereby allowing for precise prediction of treatment responses and efficacy. Preclinical drug screening based on PDLCOs models for difficult cases such as EGFR co-mutations has demonstrated promising therapeutic effects (Jia et al., 2020; Bie et al., 2021). Additionally, for patients with rare genetic mutations, such as ERBB2 mutations and RET fusions (Kim et al., 2021; Yang et al., 2020; Subbiah et al., 2018), PDLCOs can rapidly and precisely perform drug screening to guide treatment. Beyond guiding treatment for difficult and rare mutations, In addition to guiding the treatment of rare mutation and difficult patients, Chen et al. (2020) conducted drug screening on 26 common chemotherapy and targeted therapy agents in PDLCOs, revealing the solid and reliable value of PDLCOs in personalized lung cancer treatment. Furthermore, Wang et al. (2023) established an PDLCOs biobank comprising 214 samples from 107 patients and conducted a real-world study on the drug sensitivity of PDLCOs and the consistency with clinical efficacy in advanced lung cancer. The results showed that the overall accuracy of clinical efficacy for targeted therapies and chemotherapy reached 83.3%. In addition to traditional chemotherapy agents (platinum-based), targeted therapies [erlotinib (Banda et al., 2020)], and immunotherapies [pembrolizumab, nivolumab (Takahashi et al., 2019)], Li YF. et al. (2020) also explored natural compounds (chelerythrine chloride, cantharidin, harmine, berberine, and betaine) for potential breakthroughs. Drug screening experiments conducted using a biobank of PDLCOs from 10 patients showed that the organoids were more sensitive to berberine (95% CI, 0.092–1.55 μM) compared to traditional cell lines, which exhibited resistance. Although PDLCOs technology holds great potential for clinical applications, challenges remain. Currently, there is no internationally standardized protocol or technical process (Marsee et al., 2021), leading to discrepancies in research methods among different teams and difficulty in systematically simulating the progression of lung cancer and the interaction of non-tumor cells in the TME (Srivastava et al., 2020). In future lung cancer translational research, organoid technology will contribute to deepen the understanding of the multi-dimensional features and complex mechanisms of different lung cancer patients, thus facilitating more precise management and personalized clinical efficacy prediction for lung cancer patients.

### 3.2 Drug screening after resistance

PDLCOs not only provide targeted guidance for personalized treatment but also hold significant clinical value in post-resistance cases. In the treatment of lung cancer, platinum-based doublet chemotherapy remains a cornerstone of adjuvant therapy. However, genetic analysis often falls short in predicting patient responses to chemotherapy (Masters et al., 2016). Li et al. (2021) successfully developed platinum-resistant LUSC organoids and, using a combination of RNA sequencing and bioinformatics techniques, identified Halofuginone as a compound that enhances platinum drug sensitivity by inhibiting the PI3K/AKT and MAPK pathways. Over the past decade, research on EGFR-TKIs resistance in lung cancer has continued to progress (Neel and Bivona, 2017; Gazdar, 2009; Sequist et al., 2011; Chan and Hughes, 2015; Stewart et al., 2015; Cardona et al., 2017; Bartholomew et al., 2017), and it remains a major challenge in clinical treatment. Among patients receiving EGFR-targeted therapy, approximately 60% develop resistance, primarily due to secondary mutations in exon 20, particularly the T790M mutation (Metro, 2018). Resistance may also arise from non-EGFR-dependent mechanisms, including mutations in PIK3CA, BRAF, HER2, KRAS, STAT3, or XSL kinases, as well as amplifications in MET, EGFR, or CRKL (Neel and Bivona, 2017; Sequist et al., 2011; Chan and Hughes, 2015; Cardona et al., 2017). In long-term cultured Erlotinib-resistant PDLCOs, co-mutations associated with resistance, such as BRAF V600E, KRAS G12D, KRAS G12V, and PIK3CA H1047R, have been detected. These findings indicate that tumor cells often harbor multiple mutations, suggesting that combination therapies may be required for some Erlotinib-resistant patients to overcome resistance (Banda et al., 2020). However, while combination therapies may enhance efficacy, they can also lead to more severe side effects, complicating the exploration of potential drug combinations (Banda et al., 2020). Therefore, PDLCOs serve as a valuable and indispensable tool for drug screening in lung cancer patients. In PDLCOs resistant to the third-generation EGFR-TKI (osimertinib), treatment with the DCLK1 inhibitor (DCLK1-IN-1) downregulates the Wnt/β-catenin signaling pathway, thereby restoring tumor sensitivity to osimertinib (Yan et al., 2022). This offers new therapeutic prospects for patients with resistance to third-generation EGFR-TKIs. Overall, in the comprehensive management of lung cancer treatment, especially when patients in advanced stages experience disease progression and treatment resistance, PDLCOs can rapidly and accurately perform drug screening, guiding post-resistance treatment, and potentially inhibiting cancer spread and metastasis, thereby improving overall survival in patients.

### 3.3 Organoid biobanks and new drug development in lung cancer

PDLCOs not only allow for screening and guiding treatment from common drug biobanks, but also serve as a platform for discovering new anticancer targets and developing drugs, particularly through large-scale drug screening after the creation of organoid biobanks. As preclinical models closely reflecting patient characteristics, PDLCOs have demonstrated the anticancer efficacy of MFF(D) 8–11 peptide mimetics and CKD9 inhibitors (SNS032, LY2857785, and AZD4573) (Seo et al., 2019; Padmanabhan et al., 2021), highlighting their potential in drug development. AXL, a member of the Tyro3-AXL-Mer (TAM) receptor tyrosine kinase (RTK) family, has emerged as a promising therapeutic target for lung cancer (Zhang et al., 2018; Linger et al., 2010) frequently overexpressed in metastatic tumors, AXL is strongly associated with resistance and poor survival outcomes (Bae et al., 2015; Byers et al., 2013; Wu et al., 2014). Taverna et al. (2020) found that testing AXL and JAK1 inhibitors (TP-0903) in PDLCOs for personalized therapy provided strong predictive information. Della Corte et al. (2019) discovered that dual inhibition of MEK and PD-L1 in organoid-based *in vitro* models enhances anti-tumor immune responses (by better recruiting immune cells to the tumor site), showing synergistic anti-tumor activity of these two targets. Furthermore, Chen et al. (2022) utilized PDLCOs models to demonstrate that reactive oxygen species (ROS) play a pivotal role in epithelial-mesenchymal transition (EMT) and cell invasion and migration. The small molecule drug Fumonisin inhibits this malignant progression by reducing cytoplasmic ROS and suppressing the AKT-mTOR signaling pathway. Notably, approximately 85% of preclinical drugs entering oncology clinical trials fail to meet the safety or efficacy standards required for regulatory approval (DiMasi et al., 2013; Arrowsmith and Miller, 2013). The use of PDLCOs in large-scale prospective clinical cohorts facilitates the integration of molecular biological features and treatment responses in tumor patients, thus accelerating the transition from clinical to laboratory stages and establishing a precision oncology platform (Bironzo et al., 2022). An increasing number of clinical trials based on PDLCOs are being registered, with over four large-scale trials (with more than 100 cases) already underway (NCT03453307, NCT03655015, NCT05092009, and NCT05136014) (Li et al., 2023). With the rapid development of bioinformatics and biomedicine, numerous treatment targets for lung cancer patients are being discovered. Establishing a biobank of drug-resistant PDLCOs encompassing various subtypes can significantly reduce clinical decision-making time for managing the same drug-resistant subtypes in clinical practice. Moreover, with the increasing rigor of ethical reviews in animal experiments and the significant material and time investments required for clinical trials, the establishment of PDLCOs biobanks enables faster, safer, and more effective drug screening to identify beneficial treatments for patients and bring them into clinical practice.

## 4 Cross-application of PDLCOs and cutting-edge disciplines

**FIGURE 3:**
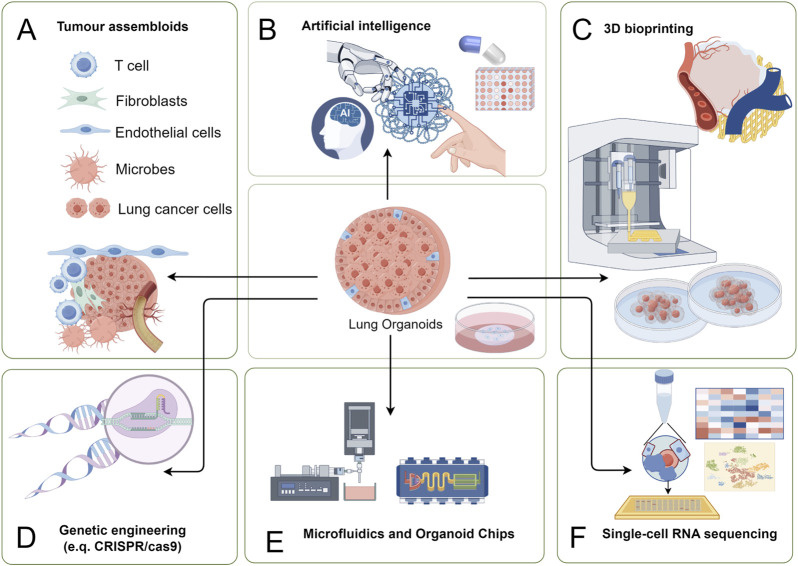
Multidisciplinary applications of PDLCOs with cutting-edge technologies. **(A)** Tumor assembloids: Co-culture of T cells, fibroblasts, endothelial cells, microbes, and lung cancer cells to construct organoid-microenvironment complex models for studying TME interactions and cancer progression; **(B)** Artificial intelligence (AI): Integration of AI algorithms for big data analysis, improving organoid data processing efficiency and optimizing personalized therapeutic strategies; **(C)** 3D bioprinting: Precise construction of organoid structures using 3D printing technology to replicate *in vivo* tissue morphology and function; **(D)** Genetic engineering: Application of CRISPR/Cas9 and other tools to edit genes, enabling studies on cancer driver genes and drug resistance mechanisms; **(E)** Microfluidics and organoid chips: Use of microfluidic chip technology to provide platforms for high-throughput drug screening and real-time observation of tumor responses; **(F)** Single-cell RNA sequencing: Single-cell sequencing to analyze cellular heterogeneity within organoids, uncovering molecular mechanisms of lung cancer.

The progress of PDLCOs research should not be confined solely to model establishment. Rather, it should involve deeper integration with cutting-edge disciplines based on stable model development. Traditional organoid technologies largely depend on the cultivation expertise of specialists in the field. However, under the context of emerging technologies, PDLCOs should explore high-throughput, standardized co-culturing methods using diverse tissue sources to better replicate the TME and the complex internal environment of the human body, thus broadening their application potential. This review further discusses the deep integration and application of PDLCOs with advanced biological and engineering technologies, including tumor assembloids technology, AI, 3D bioprinting, microfluidics and organoid-on-a-chip, gene editing, and scRNA-seq (Figure 3).

### 4.1 PDLCOs and tumor assembloids

Tumor assembloid technology emphasizes the interactions between different cell types in the TME, particularly between cancer cells and tumor-associated stromal cells, with the aim of constructing preclinical models that closely mimic the true TME (Kim et al., 2020). Currently, the application of assembloid technology in PDLCOs primarily involves co-culturing immune cells and endothelial cells. The award of the 2018 Nobel Prize in Physiology or Medicine marked a revolutionary breakthrough in cancer immunotherapy, leading to a surge in research on the impact of immunotherapy on lung cancer. Organoids models containing anti-cancer cells from the TME, such as lymphocytes, immune cells, and fibroblasts, have been widely reported (Scognamiglio et al., 2019; Holokai et al., 2020; Nakamura et al., 2019). For example, Yuki et al. (2020) demonstrated that adding exogenous immune cells to a PDLCOs culture system could reconstruct the tumor’s immune microenvironment. Cattaneo et al. (2020) successfully developed a protocol for co-culturing NSCLC organoids with lymphocytes derived from patient peripheral blood. Neal et al. (2018) utilized ALI technology to culture NSCLC organoids that retained the original TME, successfully simulating immune checkpoint blockade and restoring the anti-tumor activity of tumor-infiltrating lymphocytes (TILs). Although the survival time of TILs in this culture condition was limited (less than 60 days), the study demonstrated the potential of PDLCOs for screening immunotherapy drugs. Tumor assembloid technology has also been applied in the vascularization of the lung cancer microenvironment. Paek et al. (2019) used organoid-on-a-chip technology to construct vascularized LUAD organoids, showing that the presence of a vascular system significantly affected cell survival and vascular structure within the PDLCOs. These findings highlight the immense potential of tumor assembloids in simulating complex TME and advancing drug development. At the same time, compared to traditional organoid culture technologies, tumor assembloids are more innovative and reductive. However, standardized tumor assembloid models are still challenging to establish with high quality. The clinical application of lung cancer assembloid models requires further reproduction of the complex structures of various lung cancer types and their TME, such as complete immune cell populations, microvascular tumor infiltration, and dynamic conditions for tumor nutrition. Additionally, the effects of ionic elements and microbiota need further investigation and integration to achieve a more authentic representation of the lung cancer development process (Mai et al., 2017; Dohlman et al., 2022).

### 4.2 PDLCOs and AI

AI is profoundly transforming the landscape of biomedical research, with deep learning emerging as a key subfield that is evolving rapidly. AI methods and models have been increasingly applied across disciplines such as pathology, radiomics, genomics, and proteomics, enabling comprehensive involvement in patient diagnosis and treatment, as well as aiding in the development of personalized treatment plans (Zhu et al., 2024; Kim et al., 2024). At present, the morphological validation and high-throughput drug screening of PDLCOs primarily rely on the expertise of skilled professionals for identification and processing. The application of AI technology to process the large datasets generated by organoids can enhance the efficiency of foundational and repetitive tasks, enable deeper data mining, drive the automation as well as the standardization of organoid culture, ultimately promoting clinical translation and precision medicine (Gritti et al., 2021; Abdul et al., 2022).

In the field of lung cancer, AI algorithms have already been integrated with radiomics and clinical characteristics of patients to accurately predict overall survival and progression-free survival in NSCLC patients (Lian et al., 2022). In specific studies, Deben et al. (2023) developed an automated, high-throughput compatible live-cell imaging analysis software, OrBITS, which allows for dynamic monitoring of organoids. By combining computer vision with convolutional neural networks, OrBITS uses bright-field imaging to detect and track the growth and death of organoids. OrBITS has been validated as a useful tool for dynamic imaging and automated analysis of organoids, demonstrating superior drug screening capabilities compared to existing standard methods (CellTiter-Glo 3D assays). In drug screening for standard treatment options in lung cancer and pancreatic cancer, OrBITS not only revealed the mechanisms of drug action but also standardized drug response indicators through growth rate analysis, thereby improving the accuracy and consistency of drug response quantification. This platform provides a novel tool for the application of PDLCOs in drug development and personalized treatment, offering extensive research and clinical potential. Overall, AI technology enhances the throughput of data and the objectivity of technical processing in organoid research. However, several limitations remain. For instance, the growth of PDLCOs and the availability of high-quality image data are essential for machine learning-based modeling. Currently, most models’ predictive accuracy is assessed primarily through receiver operating characteristic curves, which may not fully and objectively reflect the actual performance of the models. Moreover, current applications primarily focus on the combination of high-throughput imaging and analysis technologies. It is anticipated that AI technology will enable a more precise integration of high-throughput imaging and analytical techniques.

### 4.3 PDLCOs and 3D bioprinting

3D bioprinting is a tissue engineering technology that allows for the precise positioning and rapid assembly of specialized biomaterials through software control. In recent years, this technology has been widely applied in the biomedical field (Mei et al., 2024; Li S. et al., 2024). By utilizing hydrogel-based designs to print appropriate biomaterials, 3D bioprinting enables a mechanized and standardized process for organoid construction (Murphy et al., 2020; Dabbagh et al., 2021). As a result, 3D bioprinting shows tremendous potential in high-throughput drug screening and the standardized construction of complex organoids. The synergistic application of PDLCOs and 3D bioprinting can lead to the development of more complex and refined cancer models, whose structures are better suited for organoid growth and maturation (Correia Carreira et al., 2020; Datta et al., 2020). Furthermore, 3D bioprinting not only enables large-scale enhancement of drug screening throughput, but also allows the simulation of complex structures incorporating various cell types, vascular systems, and even components of the nervous and immune systems. This has greatly advanced research into the potential mechanisms of TME interactions within the tumor stroma and across different tumor types (Maloney et al., 2020; Qu et al., 2021; Choi et al., 2023). Therefore, 3D bioprinting technology is highly compatible with PDLCOs in the fields of drug screening, immune microenvironment construction, and vascularization. Clark et al. (2022) innovatively developed an organoid-immersion bioprinting method, which was used for drug screening in a LUAD brain metastasis model. The combination of this rapid and accurate 3D bioprinting technique with high-throughput drug screening fully demonstrates the efficiency and rapidity of organoids in the clinical application for advanced lung cancer patients. Through 3D bioprinting, researchers can build PDLCOs models more comprehensively and rapidly, providing strong technical support for the development of personalized treatment strategies.

### 4.4 PDLCOs and microfluidic technology with organoid-on-a-chip

Organoid-on-a-chip technology is an extension of organoid techniques within the field of biotechnology. Microfluidic organoid chips, by controlling fluid perfusion, tensile forces, and chemical gradients, promote the functional maturation of organoids within a microenvironment, offering advantages such as high throughput, controllability, and dynamic continuous monitoring (Park et al., 2019). Traditional organoid culture techniques are limited by poor reproducibility, low throughput, and inadequate tissue simulation. Microfluidic organoid chips effectively address these limitations, enhancing both construction success rates and the accuracy of drug screening, thus broadening their potential applications (Park et al., 2019; Man et al., 2024). By integrating organoid culture systems with Organ-on-a-Chip technology (Wang et al., 2020), patient-derived tumor cells were combined with growth microenvironments to create a novel organoid culture model. This model can simulate both physiological homeostasis and the complexities of disease processes (Ma C. et al., 2021). Shirure et al. (2018) developed a microfluidic platform for organoids-on-a-chip, successfully constructing this culture system for further investigation into chemotherapy and anti-angiogenic treatment responses in cell lines and PDLCOs. Tan et al. (2022) used 3D bioprinting technology to successfully create a PDMS PDLCOs chip, which was co-cultured with endothelial cells. The results showed that this 3D PDLCOs chip can better mimic a pathologically relevant microenvironment. It supports multidimensional screening of single and combination drug therapies, providing real-time dynamic monitoring of therapeutic effects. This is of significant value for the personalized clinical application of EGFR-targeted therapies. While PDLCOs technologies are widely applied to advanced cancer patients, the overall survival of the patient must be considered. Although current organoid technologies can precisely identify effective therapeutic strategies, the ability to obtain results within the patient’s survival period is a critical factor for clinical applications. Hu et al. (2021) developed a superhydrophobic micro-pore array chip to generate PDLCOs from lung cancer specimens obtained through surgical resection or biopsy. This method, using relatively small sample volumes (nano-scale) and short timelines (1 week), achieved 100% accuracy in drug sensitivity testing. Additionally, PDLCOs chips preserved via direct freezing can be prepped for subsequent drug sensitivity testing, thereby saving time in high-throughput drug screening for PDLCOs (Liu et al., 2021). Therefore, organoid chip technology holds great potential for clinical use, but the overall design, material selection, and testing criteria for organoid chips must be standardized. Whether different chip materials will affect the concentration of drugs in screening or introduce potential interactions is an important issue that needs further investigation.

### 4.5 PDLCOs and gene editing

Gene editing technologies allow for the efficient and precise modification of specific targets within the genome, enabling the insertion, deletion, or replacement of genes to alter genetic information and phenotypic traits (Tanay and Regev, 2017). PDLCOs not only faithfully model the genetic and TME features of the patient’s body but also offer editability and scalability. This makes gene editing-based organoid technologies highly promising for clinical applications. Miura et al. (2021) developed an iHER2-hiPSCs model using hiPSCs and induced HER2/ERBB2 overexpression via tetracycline. The cells were differentiated into lung cancer precursor cells expressing the NKX2-1/TTF-1 lung lineage markers and co-cultured with human fetal fibroblasts to form AOs containing alveolar type II-like cells. Overexpression of HER2 in AOs resulted in the formation of tumor structures resembling atypical adenomatous hyperplasia, with enhanced proliferative abilities. Dost et al. (2020); Moye et al. (2024) utilized patient samples, genetically engineered mouse models, and organoid systems derived from mouse and human iPSCs differentiated into lung epithelial cells to investigate transcriptomic changes at the single-cell level following KRAS mutation activation. The results highlighted the value of organoid models in studying early molecular changes in KRAS-driven carcinogenesis, revealing transcriptional and proteomic differences between normal epithelial progenitor cells and early-stage lung cancer. This provided important tools for target research in KRAS-driven tumors. In the context of LUSC, Pan et al. (2023) found that KMT2D deletion could convert basal cell AOs into LUSC organoids. The loss of KMT2D remodels chromatin structure, suppresses the expression of protein tyrosine phosphatases, and leads to a significant increase in oncogenic RTK-RAS signaling, demonstrating the specific process of LUSC development. Additionally, CRISPR/Cas9 technology can be applied in PDLCOs models to knockout specific genes, providing valuable insights into the oncogenic potential of individual genes (Dekkers et al., 2020). In summary, CRISPR/Cas9-based gene reprogramming, combined with the construction of specific organoid models, provides crucial reference points for understanding the development of lung cancer and the oncogenic functions of specific genes.

### 4.6 PDLCOs and ScRNA-seq

ScRNA-seq refers to the analysis of tumor genomes and transcriptomes at the single-cell level, further advancing precision oncology (Tanay and Regev, 2017). Performing scRNA-seq on PDLCOs enables a comprehensive and accurate understanding of patient-specific clinical and biological characteristics, providing valuable decision-making insights for clinicians. Liu et al. (2024) isolated PDLCOs and their infiltrating immune cells for scRNA-seq, investigating the dynamic response of the tumor immune microenvironment to immunotherapy. This study confirmed the central role of CD8^+^ T cells in anti-tumor immunity, providing crucial insights into the molecular mechanisms of novel immunotherapies. Moreover, scRNA-seq of PDLCOs has proven to be highly beneficial for understanding the mechanisms of tumor drug resistance (Zhao et al., 2021). ScRNA-seq of organoids not only enables the analysis of the similarities between organoids and native organs at the cellular and molecular levels, but also allows for a precise understanding of the specific roles played by individual cells within the TME.

## 5 Challenges

To date, numerous laboratories around the world have developed hundreds of organoid lines. However, the overall success rate of establishing PDLCOs ranges from 15% to 80% (Ma et al., 2022), a variability likely due to the inconsistency of culture medium compositions, which may bias the successful establishment of PDLCOs from certain tumor phenotypes and/or genotypes, or lead to genomic drift (Acosta-Plasencia et al., 2024; Li et al., 2023). Additionally, the variability in the tissue digestion time and digestion solutions used for different biopsy or surgical specimens, the differing medium formulations for various histological types, and the inconsistent criteria for morphological and histological identification after successful culture all contribute to the challenges in establishing successful organoids. Therefore, systematic comparisons of cultivation protocols used by different laboratories are needed, and the development of a standardized operational manual is crucial to advancing the field of PDLCOs. Given the high heterogeneity of lung cancer, it may be challenging to use the same culture medium formulation across all cases. Thus, the choice of culture medium should be optimized according to the driver gene status, tumor pathology, and the characteristics of the TME. Future large-scale studies should focus on identifying the transcriptomic or genomic characteristics associated with successful culture, and multi-center global collaborations could accelerate the realization of this goal. As our understanding of the biological drivers of tumor heterogeneity deepens, the design of PDLCOs culture media will become more complex, but this complexity is critical for improving the success rate of organoid modeling.

The drug sensitivity testing methods for PDLCOs also require standardized procedures. Currently, the most common approach involves using ATP-based assays (CellTiter-Glo 3D Cell Viability Assay) to assess the viability of active 3D organoids, with sensitivity determined primarily by the half-maximal inhibitory concentration. This method, derived from 2D cell drug sensitivity assays, provides relatively objective results (Vlachogiannis et al., 2018; Sachs et al., 2018). However, in 3D organoids, the presence of quiescent surviving subpopulations may introduce bias into the results. Additionally, some studies suggest that drug sensitivity can also be determined by observing the number and area of surviving organoids before and after drug treatment (Yao et al., 2020). Although this method is simple, cost-effective, and has been reported to yield results comparable to the ATP assay (Yao et al., 2020), it may involve some subjective judgment. Therefore, experts in the organoid field worldwide should engage in discussions to establish standardized drug sensitivity testing and evaluation methods. Furthermore, the use of different small-molecule inhibitors and growth factors involved in key biological pathways in PDLCOs culture media may influence the organoid’s sensitivity to specific drugs. The potential impact of animal-derived matrix gels on the differentiation of PDLCOs also remains uncertain and warrants further investigation.

In addition, the growth of PDLCOs necessitates an adequate nutritional and gaseous environment to support metabolic activities. Consequently, creating a suitable and reliable vascular system model *in vitro* is essential for advancing research in PDLCOs. Wimmer et al. (2019) induced hiPSCs to differentiate into mesodermal cells, which, upon stimulation with the growth factors VEGF-A and FGF2, formed three-dimensional human vascular organoids. Following transplantation into mice, these organoids developed into a fully functional vascular system, including arteries and capillaries. In PDLCOs culture, although micro-vessels have been integrated with PDLCOs using organoid-on-a-chip technology, this process still falls short of mimicking the actual infiltration of tumor tissue by micro-vessels. Moving forward, it is expected that further integration of 3D vascular organoids with PDLCOs will allow the creation of vascularized PDLCOs that better simulate the growth and invasion processes of lung cancer.

Another challenge is the potential overgrowth of AOs, which can impact the transformation potential of PDLCOs (Karthaus et al., 2014; van de Wetering et al., 2015). It has been reported that the establishment rate of pure NSCLC organoids is only 17% due to the overgrowth of AOs (Dijkstra et al., 2020). This occurs because resected lung tumors or biopsy samples may contain retained normal airway/alveolar epithelial cells, making the presence of AOs difficult to avoid. Studies have shown that AOs are typically round (Sachs et al., 2019), while Kim et al. (2019) described AOs with a budding morphology that replicate the cellular composition and spatial architecture of normal bronchial mucosa, including p63-positive basal cells, mucin-secreting goblet cells, ciliated respiratory epithelial cells, and CC10-expressing club cells. Since TP53 mutations are present in the majority of NSCLC cases (Deben et al., 2016), some studies suggest that Nutlin-3a can drive TP53 wild-type AOs into senescence or apoptosis, thereby promoting the growth of PDLCOs with TP53 mutations. However, the use of Nutlin-3a may promote the growth of adjacent TP53-mutant airway epithelial cells, potentially causing toxicity during long-term culture (Rossi et al., 2022). Additionally, non-TP53 mutant lung cancer cells may be excluded. Apart from Nutlin-3a, PDLCOs can also be screened by removing EIF (EGF/insulin-like growth factor-1/fibroblast growth factor-2) from the culture medium and adding pan-ERBB inhibitors (Ei) to prevent the overgrowth of AOs (Ebisudani et al., 2023). Furthermore, pure PDLCOs can be established from metastatic NSCLC biopsy samples or pleural effusion specimens (Sachs et al., 2019).

Although the microbial infection rate in PDLCOs is relatively low compared to other tumor organoids, fungal, bacterial, and *mycoplasma* contamination can still occur throughout the entire process of PDLCOs cultivation. Given that lung cancer patients often have compromised immune systems, they are more prone to various pathogen-induced pneumonias (e.g., bacterial, fungal, and *mycoplasma* pneumonia). Additionally, improper handling during specimen processing, cultivation, and laboratory conditions can lead to unnecessary contamination. Therefore, it is essential to carefully observe and record the cultivation process of the PDLCOs and strictly follow standardized procedures to ensure reliable cultivation.

Finally, while the implementation of organoid-on-a-chip technology has accelerated the clinical translation of PDLCOs to less than 1 week, obtaining an adequate number of primary tumor cells from patients with advanced lung cancer through invasive procedures remains a significant challenge. In addition, physicians must make timely decisions regarding the next course of action within a limited timeframe, which requires the integration of various cutting-edge scientific approaches to maximize clinical benefits for patients. Furthermore, after determining the treatment plan for the primary tumor, it is crucial to consider whether it can also address metastatic lesions, while ensuring that the patient can tolerate the drug toxicity associated with the proposed treatment regimen.

## 6 Conclusion and future perspectives

In 2022, the FDA’s Modernization Act 2.0 explicitly proposed that the use of animal models should be gradually reduced, while promoting the adoption of alternative technologies (Adashi et al., 2023). Organoids, as preclinical models capable of highly recapitulating the genetic features and heterogeneity of patients, exhibit immense potential for application. Their unique advantage lies not only in accurately recapitulating the patient’s genetic features but also in enabling researchers to assess the impact of intercellular interactions and their spatial organization on drug responses. Compared to other types of tumor organoids, PDLCOs have a broader range of sources, including surgical resection specimens, sputum, pleural effusion, biopsy specimens, bronchoscopy specimens, and even CTCs. This diversity in sources offers greater possibilities and opportunities for the translational application of PDLCOs. Therefore, it is strongly recommended to establish personalized organoid biobanks for lung cancer patients after diagnosis. This not only supports precision medicine but also addresses challenges arising from changes in the patient’s genetic background or pathological type. Given the crucial role of complex genetic mutations in lung cancer for targeted therapies, such personalized organoid banks can guide treatment decisions even after resistance develops and support the entire process of precision treatment. Furthermore, the clinical applicability of drug screening results using PDLCOs should be continuously monitored and validated. For advanced lung cancer patients, CTCs, as key cellular populations in the metastatic process, represent high invasiveness and metastatic potential. CTCs detection can reveal tumor progression at least 1 month in advance, and PDLCOs constructed from CTCs provide more time for clinical decision-making. Given the unique immune microenvironment of lung cancer, along with its higher oxygenation and blood supply compared to other cancers, ALI co-culture methods may significantly enhance the application value of PDLCOs. This is particularly valuable for clinical research and the simulation of tumor initiation and progression mechanisms and warrants further exploration.

Currently, traditional PDLCOs culture technologies have not yet fully met the demand for rapid and precise clinical applications. Future research and applications will advance rapidly through interdisciplinary integration of multiple technologies. Engineering technologies based on organoid chips are undoubtedly set to elevate the clinical application of PDLCOs to new heights (Wu et al., 2024). To begin with, PDLCOs chips can control the culture volume at the nanoscale level, allowing for precise regulation of reagent usage and cost reduction (Schuster et al., 2020). In addition, microfluidic technology enhances the heterogeneity and stability of PDLCOs models by adjusting fluid shear forces and optimizing the concentrations of oxygen and nutrients (Li X. et al., 2020; Tao et al., 2019). Furthermore, the integration of AI technologies and high-content imaging systems enables more efficient identification of PDLCO model characteristics, thus facilitating sensitive detection and analysis of TME and drug screening results (Mencattini et al., 2020). Finally, PDLCOs chips can not only co-culture with multiple cell types from the TME but also integrate organoids derived from other organ, thereby simulating the human *in vivo* environment more accurately (Paek et al., 2019; Xu et al., 2013). In addition to predicting the therapeutic effects of drugs on tumor cells, they can also forecast potential damage to other organs, thus preventing fatal side effects. The combination of PDLCOs technology, diverse specimen sources, personalized organoid bank construction, and the innovative application of organoid chips and microfluidic technologies is driving a transformation in precision treatment and clinical decision-making. This provides powerful tools and new opportunities for precision diagnosis, treatment, and resistance mechanism research in lung cancer patients.

In conclusion, PDLCOs, as rapidly evolving preclinical models, hold tremendous potential for clinical translation and offer broad prospects for both basic research and clinical treatment of lung cancer. Although current PDLCOs cannot yet fully replicate the complex pathological and physiological processes *in vivo*, nor the complete absorption and metabolism of drugs within the body, the rapid advancement of cutting-edge multidisciplinary fields suggests that the development of comprehensive, personalized PDLCOs systems is expected to expedite their translation from laboratory to clinical applications.

## Abbreviations

PDLCOs, Patients derived lung cancer organoids; LUAD, Lung adenocarcinoma; LUSC, Lung squamous cell carcinoma; NSCLC, Non-small cell lung cancer; AOs, Airway organoids; SCLC, Small cell lung cancer; PDX, Patient-derived xenograft; ALI, Air-liquid interface; TGF-β, transforming growth factor beta; EMT, epithelial mesenchymal transition; WES, whole-exome sequencing; IF, immunofluorescence; IHC, Immunohistochemistry; HE, Hematoxylin and eosin; WGS, Whole genome sequencing; MSE, malignant serous effusion; TKI, tyrosine kinase inhibitor; TME, tumor microenvironment; 2D, two-dimensional; 3D, three-dimensional; ECM, extracellular matrix; CTCs, circulating tumor cells; hiPSCs, human-induced pluripotent stem cells; scRNA-seq, single cell RNA sequencing.
