# Supplementary material for: Bridging the gap: how patient-derived lung cancer organoids are transforming personalized medicine
Source: Front Cell Dev Biol. 2025 Apr 15;13:1554268. doi: 10.3389/fcell.2025.1554268 (PMC12037501; doi:10.3389/fcell.2025.1554268)
Supplement: Supplementary file 2 [file Table2.docx]

TableS2.Organoid culture using hydrogels from different sources

| Matrix Gel Type | Characteristics (Optimized) | References |
| --- | --- | --- |
| **Natural Hydrogels** | Composed of natural polymer-based materials containing protein fiber networks and glycosaminoglycans. |  |
| Matrigel | Composite of ECM proteins, proteoglycans, and growth factors secreted by EHS mouse sarcoma cells. | PMID:39236699, PMID: 36806896, PMID: 38773241 |
| Plant Nanocellulose Hydrogels | Cellulose-based hydrogels where cellulose serves as mechanical scaffolding, supplemented with physicochemical signals to support cellular growth. | PMID: 33437574, PMID: 35304464 |
| Fibrin Hydrogels | Polymer gel formed through non-covalent cross-linking via proteolytic exposure of binding sites. | PMID: 30203567 |
| Hyaluronic Acid Hydrogels | Animal-free matrix formed by thiol-modified hyaluronic acid reacting with thiol-reactive crosslinkers. | PMID: 29755963  PMID: 36332268 |
| Sodium Alginate Hydrogels | Rapid non-covalent cross-linking with adjustable physicochemical properties. | PMID: 34494727 |
| **Synthetic Hydrogels** | Primarily composed of synthetic polymers, exhibiting enhanced mechanical properties but lacking bioactive components. |  |
| PEG Hydrogels | Demonstrate tunable biodegradability, precise molecular weight control, excellent mechanical properties, and facile chemical modification. | PMID: 31951973  PMID: 33334213 |
| Polyacrylamide Hydrogels | Feature superior mechanical strength, porous microstructure, thermoreversible behavior, and biological inertness. | PMID: 39044566  PMID: 34658689 |
| Polyisocyanate Hydrogels | Thermoreversible materials with non-immunogenic properties. | PMID: 36818041 |
| Self-assembling Hydrogels | Based on the self-assembly of peptides into fiber structures, a self-assembled hydrogel with a stable β-folded structure is formed. | PMID: 38657288 |
